# Effects of candesartan, an angiotensin II receptor type I blocker, on atrial remodeling in spontaneously hypertensive rats

**DOI:** 10.14814/phy2.12274

**Published:** 2015-01-27

**Authors:** Stéphanie C. Choisy, Shang‐Jin Kim, Jules C. Hancox, Sandra A. Jones, Andrew F. James

**Affiliations:** Cardiovascular Research Laboratories, School of Physiology & Pharmacology, School of Medical Sciences, University of Bristol, Bristol, U.K; Department of Pharmacology and Toxicology, College of Veterinary Medicine, Chonbuk National University, Jeonju‐City, South Korea; School of Biological, Biomedical and Environmental Sciences, University of Hull, Hull, U.K

**Keywords:** Angiotensin receptor blocker, connexin‐43, fibrosis, gap junction

## Abstract

Hypertension‐induced structural remodeling of the left atrium (LA) has been suggested to involve the renin–angiotensin system. This study investigated whether treatment with an angiotensin receptor blocker, candesartan, regresses atrial remodeling in spontaneously hypertensive rats (SHR). Effects of treatment with candesartan were compared to treatment with a nonspecific vasodilatator, hydralazine. Thirty to 32‐week‐old adult male SHR were either untreated (*n *= 15) or received one of either candesartan cilexetil (*n *= 9; 3 mg/kg/day) or hydralazine (*n *= 10; 14 mg/kg/day) via their drinking water for 14 weeks prior to experiments. Untreated age‐ and sex‐matched Wistar‐Kyoto rats (WKY;* n *= 13) represented a normotensive control group. Untreated SHR were hypertensive, with left ventricular hypertrophy (LVH) compared to WKY, but there were no differences in systolic pressures in excised, perfused hearts. LA from SHR were hypertrophied and showed increased fibrosis compared to those from WKY, but there was no change in connexin‐43 expression or phosphorylation. Treatment with candesartan reduced systolic tail artery pressures of conscious SHR below those of normotensive WKY and caused regression of both LVH and LA hypertrophy. Although hydralazine reduced SHR arterial pressures to those of WKY and led to regression of LA hypertrophy, it had no significant effect on LVH. Notably, LA fibrosis was unaffected by treatment with either agent. These data show that candesartan, at a dose sufficient to reduce blood pressure and LVH, did not cause regression of LA fibrosis in hypertensive rats. On the other hand, the data also suggest that normalization of arterial pressure can lead to the regression of LA hypertrophy.

## Abstract (precis)

Structural remodeling of the atria, involving atria enlargement and fibrosis, in hypertension increases the risk of atrial fibrillation (AF). Treatment of spontaneously hypertensive rats with the angiotensin receptor blocker, candesartan, reduced arterial pressure and myocardial hypertrophy to the level of normotensive rats but had no effect on atrial fibrosis. The resistance of hypertension‐associated atrial fibrosis to the AT1 receptor antagonist may provide insight into the basis to the ineffectiveness of drugs targeting the renin–angiotensin system in reducing incidence of AF in hypertensive patients.

## Introduction

It has been suggested that structural changes to the atria as a result of an underlying pathology, termed ‘atrial remodeling’, predispose the heart to atrial fibrillation (AF) (Casaclang‐Verzosa et al. 2008; Benjamin et al. 2009). Dilatation and enlargement of the left atrium (LA), together with left ventricular hypertrophy (LVH), are considered to be good epidemiological indicators of the risk of AF in patients (Vaziri et al. 1994; Tsang et al. 2002b). It is thought that atrial enlargement, interstitial fibrosis, and remodeling of gap junctions of the myocardium, by establishing paths of sufficient length and interfering with normal conduction, contribute to a pro‐arrhythmic substrate favouring reentry (Spach and Boineau 1997; Nattel 2002; Severs et al. 2008). Evidence from animal models involving chronic rapid pacing of the atria indicates that the existence of AF itself leads to atrial structural remodeling stabilizing the arrhythmia (‘AF begets AF’) (Wijffels et al. 1995; Ausma et al. 1997; Schotten et al. 2003). However, the majority of patients with AF (>70%) have some form of preexisting structural heart disease (e.g., heart failure, valve dysfunction, and hypertension) and it is supposed that these pathologies give rise to the pro‐arrhythmic substrate in which AF is initiated (Casaclang‐Verzosa et al. 2008; Benjamin et al. 2009). The most prevalent independent risk factor for AF is the existence of systemic hypertension and there is a clear relationship between arterial blood pressure, LVH, and LA enlargement (Benjamin et al. 1994; Vaziri et al. 1995). Data from animal models of systemic hypertension have not only strengthened the case for elevated arterial pressure as a cause of atrial remodeling but also provide further evidence supporting an association between atrial enlargement, interstitial fibrosis, and susceptibility to AF (Kistler et al. 2006; Choisy et al. 2007).

The mechanisms linking hypertension to atrial remodeling remain unclear but it has been proposed that chronically elevated ventricular afterload results in hemodynamic overload of the left atrium, presumably leading to the activation of stretch‐induced signaling pathways in the atrial wall (Vaziri et al. 1995; Tsang et al. 2002a; Casaclang‐Verzosa et al. 2008). Activation of the renin–angiotensin–aldosterone system (RAAS) has also been suggested to contribute to atrial remodeling (Healey et al. 2005b). In addition to its vasopressor action, angiotensin‐II, acting via AT_1_ receptors, stimulates cardiac myocyte hypertrophy, changes in myocardial expression of the gap junction protein, connexin‐43, and synthesis of collagen by cardiac fibroblasts (Sadoshima and Izumo 1993; Brilla et al. 1994; Tsai et al. 2008b; Adam et al. 2010). Infusion of experimental animals with angiotensin‐II leads to atrial remodeling involving atrial dilatation and interstitial fibrosis (Sun et al. 1997; Tsai et al. 2008b; Yagi et al. 2010). There has therefore been considerable interest in whether drugs targetted to the RAAS, such as angiotensin‐converting enzyme (ACE) inhibitors and angiotensin receptor blockers (ARB), reduce the incidence of AF or its recurrence in at‐risk patients (Healey et al. 2005a; Schneider et al. 2010; Galzerano et al. 2012). A number of studies in animal models have demonstrated the effectiveness of treatment with ACE inhibitors and ARB in the inhibition of atrial remodeling associated with the long‐term infusion of angiotensin‐II (Tsai et al. 2008b), with atrial fibrillation (Kumagai et al. 2003; Anné et al. 2007; Li et al. 2007; Tsai et al. 2008a; Liu et al. 2010) and with heart failure (Li et al. 2001; Shimano et al. 2008). However, it is striking that there are relatively few if any data on the effects of RAAS inhibition on atrial remodeling in animal models of hypertension. This study was conducted in order to examine the effectiveness of treatment with candesartan, in comparison with the nonspecific vasodilator hydralazine, in the regression of atrial remodeling in spontaneously hypertensive rat hearts.

## Materials and Methods

### Animals and drug administration

All procedures were conducted in accordance with the Animals (Scientific Procedures) Act 1986 of the United Kingdom and were approved by the University of Bristol Ethical Review Committee. Adult male spontaneously hypertensive rats (SHR) of 30–32 weeks of age were assigned to either control group (SHR control, *n* = 15) or to receive one of either hydralazine (SHR hydra, 14 mg/kg/day, *n* = 10) or candesartan cilexetil (SHR can, 3 mg/kg/day, *n* = 9) via their drinking water for 14 weeks prior to experiments. This dosage regime for hydralazine has previously been shown to be effective in reducing systolic arterial pressure in SHR (Kohya et al. 1995; Tsotetsi et al. 2001). The dosage regimen for candesartan cilexetil was selected on the basis of preliminary experiments to be sufficient to reduce systolic arterial pressure in SHR to or below the systolic arterial pressure of Wistar‐Kyoto normotensive controls (WKY; data not shown). Animals were 44–46 weeks of age at the time of experiments, corresponding to an age at which significant remodeling of the left atrium and increased susceptibility to atrial tachyarrhythmia had previously been established to occur in SHR hearts (Choisy et al. 2007). SHR weighed 354 ± 8 g at the time of experiments and body weight was unaffected by drug treatment (SHR hydra: 357 ± 3 g; SHR can: 323 ± 14 g). Age‐ and sex‐matched WKY normotensive controls (*n* = 13) received control treatment. WKY weighed 475 ± 13 g at the time of experiments (*P* < 0.0001 vs. untreated SHR, Bonferroni multiple comparisons post hoc test). Systolic pressure in the tail artery was measured in conscious animals during the week prior to experiments by tail cuff plethysmography (Choisy et al. 2007). Daily water consumption was monitored in order to calculate the appropriate concentration of drug to add to the drinking water in order to achieve the daily dose. Hydralazine was soluble directly in water. Candesartan cilexetil (Sequoia Research Products, Pangbourne, U.K.) was dissolved to ~10 times the final concentration in a vehicle of polyethylene glycol 400 (10% v/v), ethanol (5% v/v), cremophor EL (2% v/v), and tap water (83% v/v) and the pH was adjusted to 9.0 with 0.5 mol/L Na_2_CO_3_ prior to dilution to the final concentration in tap water, according to Seltzer et al. (2004). SHR (*n* = 7) and WKY rats (*n* = 5) were treated with vehicle alone via the drinking water but since there was no significant difference in any of the measured parameters (e.g., tail artery pressure, left ventricular weight/heart weight ratio) between vehicle‐treated and untreated age‐ and sex‐matched SHR and WKY rats, the data were combined into an SHR control group and a WKY control group.

### Whole heart perfusion

On the day of experimentation, body weight was recorded prior to sodium pentobarbital general anesthesia (i.p. 60–80 mg/kg). Hearts were isolated from the rats under general anesthesia, mounted on a whole heart perfusion apparatus and perfused retrogradely via the aorta with a Krebs’ Henseleit solution (in mmol/l; 118.5 NaCl, 25.0 NaHCO_3_, 3.0 KCl, 1.2 MgSO_4_.7H_2_O, 1.2 KH_2_PO_4_, 2.5 CaCl_2_, 11.1 d‐glucose at 37°C) gassed with 95% O_2_/5% CO_2_. As described previously, after 20 min of Langendorff perfusion, a cannula was inserted into the left atrium and orthograde perfusion established in the so‐called “working heart” mode with the preload and after‐load set to, respectively, 13 and 62 mmHg (Choisy et al. 2007). The aortic pressure, atrial pressure, and electrocardiogram (ECG) were recorded using the PowerLab 8/SP data acquisition system and Chart software version 5 (AD Instruments Ltd, Oxford, U.K.). Atrial effective period (AERP) and conduction velocity (CV) were measured by the recording of bipolar electrograms from the epicardial surface of the left atrium using a 5 × 5 electrode array as described previously (Kim et al. 2011, 2012). The inducibility of atrial tachyarrhythmias was examined by 5 sec of burst pacing at cycle lengths of <10 msec (Kim et al. 2011, 2012). At the end of experiments, hearts were removed from the perfusion apparatus, dissected, and wet heart weight, left atrial weight, and left ventricular weight recorded. Left atria were embedded in Tissue Tek OCT^®^ (Sakura Finetek Europe B.V., Alphen aan den Rijn, Netherlands) and snap frozen in preparation for histological analysis.

### Histology and immunocytochemistry

Cryo‐sections (10 *μ*m) of left atrial tissue were prepared as described previously (Jones et al. 2004). Frozen sections were fixed using 4% paraformaldehyde for 2 min and washed with PBS. Sections were dehydrated using an increasing ethanol series (70%, 85%, and 95% at −20°C). For the histological analysis of fibrosis, fixed sections were stained with Masson's trichrome, digitized images obtained, and the blue pixel content measured relative to the total tissue area using Adobe Photoshop CS2. A total connexin‐43 antibody (MAB 3068, Chemicon International Inc., Temecula, CA) and a phospho‐connexin‐43 (connexin‐43P) antibody (3511S, Cell Signaling Technology, Beverly, MA) specific to phosphorylation at serine 368 (Solan et al. 2003) were used. AlexaFluor^®^488‐conjugated anti‐mouse IgG1 secondary (A21121, Invitrogen, Paisley, U.K.) were applied at 1 *μ*g/mL. Using methods that we have reported previously (Kim et al. 2011), confocal images were collected using a LSM‐510 laser scanning microscope using the same settings (i.e., objective lens, laser power, photomultiplier gain, and pixel size) for each image (Carl Zeiss Ltd, Cambridge, U.K.). Images of equal area (95.5 × 88.9 *μ*m) were selected at random from sections (a single image per section) from control WKY (WKY, *n *= 8 sections from four rats), control SHR (SHR control, *n *= 8–9 sections from four rats), candesartan‐treated SHR (SHR can, *n *= 8–9 sections from three rats), and hydralazine‐treated SHR (SHR hydra, *n *= 8 sections from three rats) tissue sections and connexin‐43‐specific fluorescent staining quantified using ImageJ (v1.48, NIH).

### Statistics

Data are presented as mean ± SEM. Student's *t*‐test and one‐way ANOVA with Bonferroni multiple comparisons post hoc tests were performed, as appropriate, using Prism 5.03 (GraphPad Software Inc, San Diego, CA). *P *< 0.05 was considered statistically significant.

## Results

**Figure 1.**
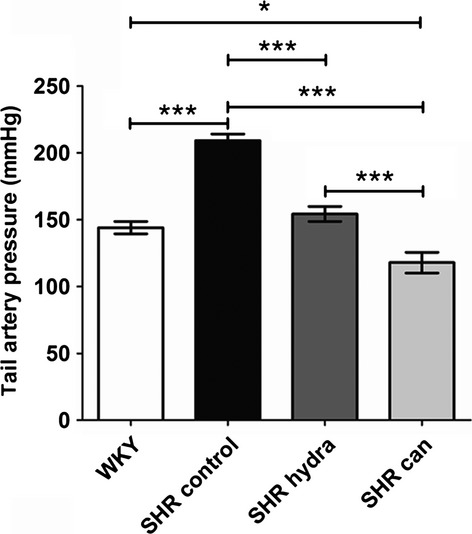
Effect of antihypertensive treatment on tail artery blood pressure in conscious rats. WKY – untreated and vehicle‐treated Wistar Kyoto rats; SHR control – untreated and vehicle‐treated spontaneously hypertensive rats; SHR hydra – hydralazine‐treated SHR; SHR can – candesartan‐treated SHR. *P < 0.05; ***P < 0.0001; Bonferroni's multiple comparisons post hoc test.

Systolic tail artery pressures were significantly higher in conscious spontaneously hypertensive rats of 44–46 weeks of age compared with those from age‐matched WKY normotensive controls (Fig. 1). Prior treatment of the hypertensive rats with oral hydralazine for 14 weeks significantly reduced tail artery pressures, effectively to the same level as normotensive controls (Fig. 1). Arterial pressures in hypertensive rats were also significantly reduced by treatment with oral candesartan for the same period of time, although in this case to a value significantly less than the systolic pressure of normotensive animals (Fig. 1).

**Figure 2.**
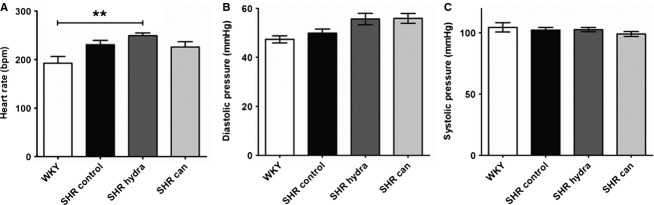
Function of excised perfused working hearts. (A) Heart rate. **P < 0.01, Bonferroni's multiple comparisons post hoc test. (B) Aortic diastolic pressure. (C) Aortic systolic pressure.

**Figure 3.**
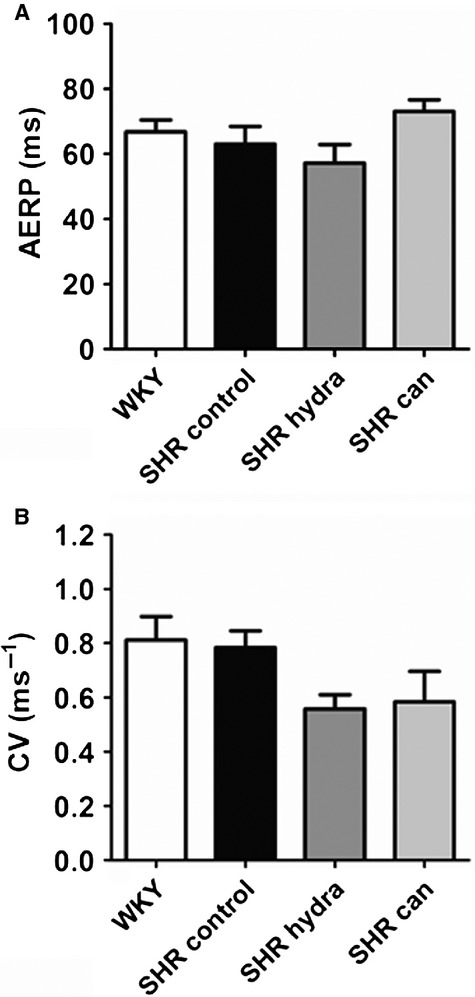
Atrial electrophysiology of perfused hearts. (A) Atrial effective refractory period (AERP). (B) Conduction velocity (CV).

The heart rate in sinus rhythm was slightly, although not significantly, greater in excised perfused hearts from hypertensive rats (SHR) compared with hearts from normotensive WKY controls (Fig. 2A). Pacemaking activity by the sino‐atrial node has been suggested to be remodeled in SHR hearts (Heaton et al. 2006; Choisy et al. 2007). Treatment with neither of the antihypertensive agents had any significant effect on heart rate in spontaneously hypertensive rat hearts (Fig. 2A). There were no significant differences between excised perfused SHR and WKY hearts in aortic diastolic (Fig. 2B) or systolic (Fig. 2C) pressures, indicating that ventricular contractility was not impaired in hearts from hypertensive rats. Although mean diastolic pressures were slightly greater in hearts from drug‐treated SHR compared with vehicle‐treated SHR hearts, this was not statistically significant (Fig. 2B). Atrial effective refractory period (AERP) and conduction velocity (CV) in perfused SHR hearts were not significantly different to perfused WKY hearts (Fig. 3A and B), consistent with previous reports (Choisy et al. 2007; Lau et al. 2013). Although mean CV in hearts from antihypertensive‐treated SHR were lower than in hearts from untreated hypertensive rats, this was not statistically significant (Fig. 3B).

**Figure 4.**
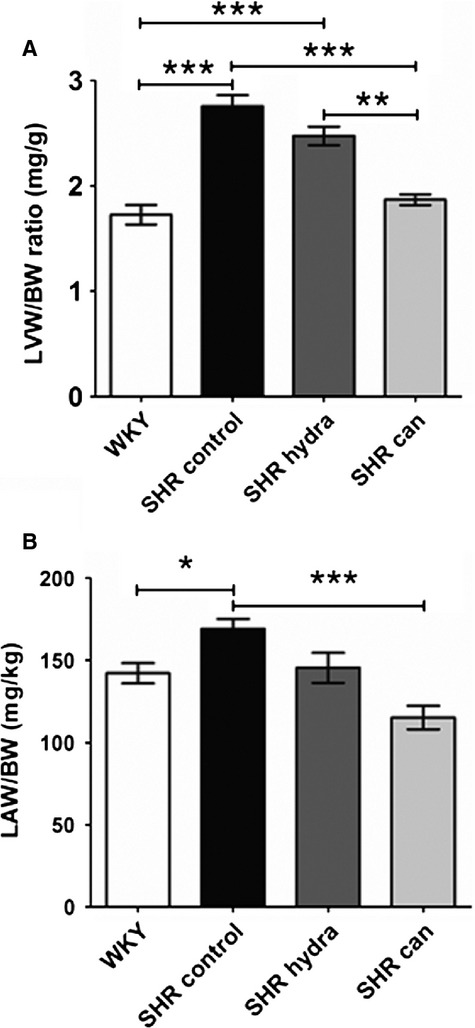
Effect of antihypertensive treatment on (A) ventricular and (B) atrial hypertrophy. *P < 0.05; **P < 0.01; ***P < 0.0001; Bonferroni's multiple comparisons post hoc test.

Hearts from hypertensive rats showed significant left ventricular hypertrophy (LVH) compared with hearts from the normotensive WKY controls, the mean left ventricular weight/body weight (LVW/BW) ratio of SHR hearts being 60% greater than that of WKY hearts (Fig. 4A). Reduction of arterial pressure in SHR to normotensive levels with hydralazine did not lead to significant regression of the LVH (Fig. 4A). In contrast, treatment of hypertensive rats with candesartan effectively reduced the LVW/BW ratio to the same level as in hearts from age‐matched normotensive animals (Fig. 4A). This indicates that angiotensin‐II type 1 (AT_1_) receptor blockade, but not normalization of systolic arterial pressure, resulted in regression of LVH. In addition to LVH, hypertension was also associated with left atrial hypertrophy (LAH) as the left atrial weight/body weight (LAW/BW) ratio of SHR hearts was approximately 20% greater than that of hearts from WKY normotensive controls (Fig. 4B). In contrast to the effects on the LVH, treatment of SHR with either candesartan or hydralazine reduced LAW/BW ratios to values not significantly different from WKY (Fig. 4B).

**Figure 5.**
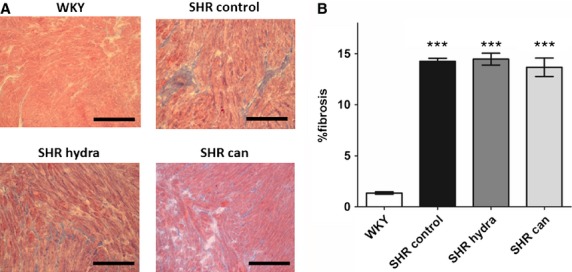
Effect of antihypertensive treatment on left atrial fibrosis. (A) Representative photomicrographs of Masson's trichrome‐stained slides of left atrial tissue. Scale bars represent 100 *μ*m. (B) Mean (±SEM) percentage fibrosis in Masson's trichrome‐stained slides of left atrial tissue. ***P < 0.0001; Bonferroni's multiple comparisons post hoc test, versus WKY.

**Figure 6.**
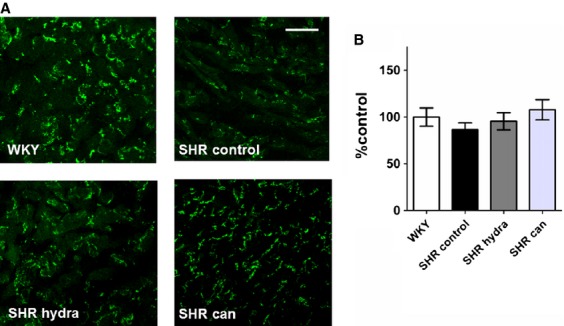
Effect of antihypertensive treatment on total connexin‐43 expression. (A) Representative sections of anti‐connexin‐43‐stained sections of left atrial tissue. Scale bars represent 50 *μ*m. (B) Mean (±SEM) connexin‐43 expression calculated as percentage fluorescence intensity relative to mean WKY‐control level.

**Figure 7.**
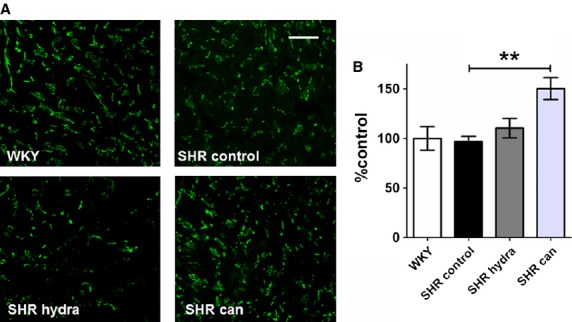
Effect of antihypertensive treatment on phosphorylated connexin‐43 (connexin‐43P) expression. (A) Representative sections of anti‐connexin‐43P‐stained sections of left atrial tissue. Scale bars represent 50 *μ*m. (B) Mean (±SEM) connexin‐43P expression calculated as percentage fluorescence intensity relative to mean WKY control level. **P < 0.01; Bonferroni's multiple comparisons post hoc test, versus SHR control

Left atrial sections from SHR hearts showed marked interstitial fibrosis compared with sections from normotensive control hearts (Fig. 5A). The cell edges and intercalated disks could not reliably be identified in these sections and it was not possible, therefore, to assess whether the reduction of left atrial hypertrophy on treatment with the antihypertensive agents reported in Figure 4 was associated with a reduction in cell size. Nevertheless, treatment with neither hydralazine nor candesartan resulted in regression of fibrosis in the left atria of spontaneously hypertensive rat hearts (Fig. 5B). Since changes in connexin‐43 expression and in phosphorylation of connexin‐43 at serine 368 have been implicated in pro‐arrhythmic remodeling in heart failure (Burstein et al. 2009), the remodeling of connexin‐43 and the effects of the antihypertensive drugs were investigated. In contrast to the fibrosis, there were no detectable differences in either the pattern or level of expression of connexin‐43 and phosphorylated connexin‐43 between left atrial sections from SHR hearts and normotensive WKY controls (Figs. 6, 7). Curiously, treatment of SHR with candesartan resulted in significantly greater signal for phosphorylated connexin‐43 compared with control‐treated SHR (Fig. 7B).

## Discussion

This study presents novel evidence that the AT_1_ receptor antagonist candesartan, at doses sufficient to reduce systolic arterial pressure and produce regression of left ventricular hypertrophy, does not result in regression of left atrial fibrosis in spontaneously hypertensive rats. On the other hand, reduction of arterial pressure by either candesartan or the non‐specific vasodilatator, hydralazine, was associated with regression of left atrial hypertrophy. The regression of LVH in SHR by treatment with candesartan, but not by hydralazine, in this study is consistent with a previous report that the simple lowering of arterial pressure was insufficient to cause regression of LVH but that AT_1_ receptor blockade using candesartan had a significant antihypertrophic effect (Kohya et al. 1995). Moreover, the data demonstrate that the dose of candesartan used was effective in the regression of ventricular remodeling, presumably through actions at ventricular AT_1_ receptors (Kohya et al. 1995). Our data demonstrating left atrial weight/body weight ratios in both candesartan‐ and hydralazine‐treated SHR that were not significantly different to the normotensive WKY control suggest that the reduction in arterial pressure is sufficient to reverse the atrial hypertrophy in this model, but not the interstitial fibrosis, and are consistent with distinct signaling pathways underlying the hypertrophic and the fibrotic responses to hypertension (Burstein and Nattel 2008).

### Gap junctions

In contrast to previous reports from models of heart failure and surgical aortic stenosis in rats, there was no remodeling of connexin‐43 expression in the hypertensive hearts (Rucker‐Martin et al. 2006; Kim et al. 2011; Yoon et al. 2013). It was not possible to determine the localization of connexin‐43 staining in this study and thus we were unable to assess whether there was any redistribution of gap junction proteins to the lateral membranes, as has been suggested in other models of structural heart disease (Rucker‐Martin et al. 2006; Burstein et al. 2009; Yoon et al. 2013). There was no change in phosphorylation at S368 of connexin‐43 in the atria from the hypertensive hearts, which is in contrast to a previous report from a canine model of congestive heart failure (Burstein et al. 2009). It has been suggested that the expression ratio of connexin‐40 to connexin‐43 in the atria is increased in heart failure (Burstein et al. 2009). However, since there is relatively little atrial expression of connexin‐40 in the rat heart (Gros et al. 1994; Polontchouk et al. 2001), it was not possible to examine remodeling of this gap junction protein in hypertension in this study. While the reason for the difference between the studies remains unclear, it is possible that the increase in hemodynamic load on the left atrium in this study was not sufficient to cause gap junction remodeling. In any case, the lack of changes in connexin‐43 and connexin‐43P staining is consistent with the absence of significant conduction velocity slowing in the atria of hypertensive hearts compared with normotensive controls.

Treatment of the hypertensive rats with candesartan unexpectedly resulted in an increased level of staining with the connexin‐43P antibody, suggesting an increase in the level of atrial connexin‐43 phosphorylation at serine 368 in the ARB‐treated SHR compared to vehicle‐treated SHR. The mechanism underlying this effect remains unclear; as serine 368 is a site for protein kinase C*ε*‐dependent phosphorylation, antagonism of the G_q_‐coupled AT_1_ receptor might have been expected to reduce connexin‐43 phosphorylation (Lampe et al. 2000; Ek‐Vitorin et al. 2006; Michel et al. 2013). However, AT_1_ receptors are present in many tissues and cell types, including the brain and autonomic nervous system, so that the effect of treatment with candesartan on atrial connexin‐43 phosphorylation may have been through an indirect mechanism (Michel et al. 2013). Additionally, AT_1_ receptors have been shown to couple to Rac1/STAT3 signaling pathways regulating gene expression and protein synthesis in atrial myocytes (Tsai et al. 2008b), so that the effect of candesartan in this study may have been through a change in the expression of proteins regulating connexin‐43 phosphorylation.

### Atrial fibrosis

Our results are in contrast to a previous study of atrial remodeling in rats made hypertensive by administration of the nitric oxide synthase (NOS) inhibitor, nitro‐*ω*‐l‐arginine‐methyl‐ester (l‐NAME) via the drinking water for 8 weeks (Okazaki et al. 2006). Although, as found in this study, oral treatment with hydralazine was without effect on atrial fibrosis, treatment with candesartan at a dose (0.1 mg/kg/day) lower than that used in this study significantly reduced the area of interstitial fibrosis (Okazaki et al. 2006). It is possible that the signaling processes underlying atrial fibrosis in the NOS inhibition model differ from those underlying the fibrosis in the atria of SHR, such that AT_1_ receptors contributed to the former and not to the latter remodeling and candesartan coadministration with l‐NAME sufficed to inhibit the development of the fibrosis. Oral treatment with the ARB, losartan, has also been shown to be effective in reducing the atrial fibrosis in a coronary artery ligation model of heart failure in rats (Yoon et al. 2013). In that study, the administration of losartan was started from the time of surgery so that the ARB was able to inhibit the development of fibrosis and atrial remodeling (Yoon et al. 2013). We have previously shown that, in contrast to these two studies, fibrosis of the left atria in spontaneously hypertensive rats is evident as early as 12–14 weeks of age (Choisy et al. 2007). Thus, our data suggest that oral treatment with candesartan is not effective in the regression of the atrial fibrosis induced by long‐term systemic hypertension in rats.

### Substrate for arrhythmia

Atrial interstitial fibrosis, and associated conduction abnormalities, have previously been suggested to contribute to a substrate for atrial tachyarrhythmia/atrial fibrillation in SHR hearts (Choisy et al. 2007; Lau et al. 2013). Therefore, the effects of candesartan and hydralazine treatment on the inducibility of atrial tachyarrhythmias following burst pacing would have been of considerable interest. However, in contrast to previous studies in which atrial tachyarrhythmias could be induced in 83% of hearts (Choisy et al. 2007), in this study it was only possible to induce tachyarrhythmias in three of the 15 hearts (20%). The low inducibility of atrial tachyarrhythmias is likely related to the small size of the left atria of SHR hearts in this study (60 ± 3 mg) as compared with our previous study (98 ± 7 mg, *n *= 22; *P* < 0.0001, Student's unpaired *t*‐test) and as compared with WKY in this study (67 ± 3 mg, *n *= 13). The small size of the atria of SHR hearts in this study is associated with low body weight as compared with the previous study (354 ± 8 g vs. 456 ± 9 g; *P* < 0.0001, Student's unpaired *t*‐test). Thus, it seems likely that the size of the substrate, in addition to the existence of fibrosis and conduction abnormalities, plays an important role in the inducibility of tachyarrhythmia. Moreover, as a consequence of the low number of SHR hearts in which atrial tachyarrhythmias could be induced, this study was not sufficiently statistically powered to assess the effect of the antihypertensive treatment on the inducibility of arrhythmias.

Nevertheless, our findings may provide some insight into the lack of effectiveness of ACE inhibitors and ARB in the reduction of AF incidence in post hoc analyses of trials in patients with primary hypertension (Healey et al. 2005b; Schneider et al. 2010). While the Losartan Intervention For Endpoint Reduction in Hypertension (LIFE) and Valsartan Antihypertensive Long‐term Use Evaluation (VALUE) studies have suggested a reduction in new onset AF with AT_1_ receptor blockade (Wachtell et al. 2005; Schmieder et al. 2008), other trials involving ACE inhibitors (e.g., Hansson et al. 1999a,b; Salehian et al. 2007) or ARB (e.g., Yusuf et al. 2008) have not revealed any benefit of RAAS inhibition in patients with primary hypertension. A meta‐analysis of these trials indicates no overall benefit of ACE inhibitors or ARB in the reduction of AF in hypertensive patients (Schneider et al. 2010). Our data support the view that while these agents are effective antihypertensives, they may not be able to regress the interstitial fibrosis that plays an important role in the substrate for arrhythmia in hypertension (Nattel and Opie 2006). A more complete understanding of the mechanisms underlying atrial remodeling in hypertension is warranted in order to identify novel potential therapeutic targets for the regression of atrial fibrosis; studies using animal models of hypertension, such as the spontaneously hypertensive rat, are likely to be highly valuable in this regard.

## Acknowledgments

The authors wish to thank Mrs Lesley A. Arberry for technical assistance.

## Conflicts of Interest

The authors have no conflicts of interest to declare.
